# The incidence of and risk factors for hospitalisations and amputations for people with diabetes‐related foot ulcers in Queensland, 2011–19: an observational cohort study

**DOI:** 10.5694/mja2.52703

**Published:** 2025-07-13

**Authors:** Yuqi Zhang, Susanna M Cramb, Steven M McPhail, Rosana Pacella, Jaap J van Netten, Ewan M Kinnear, Peter A Lazzarini

**Affiliations:** ^1^ Australian Centre for Health Services Innovation and Centre for Healthcare Transformation Queensland University of Technology Brisbane QLD; ^2^ Karolinska Institute Stockholm Sweden; ^3^ Jamieson Trauma Institute, Metro North Hospital and Health Service Brisbane QLD; ^4^ Metro South Health Brisbane QLD; ^5^ Institute for Lifecourse Development, University of Greenwich London United Kingdom; ^6^ University of Amsterdam Amsterdam The Netherlands; ^7^ The Prince Charles Hospital, Metro North Hospital and Health Service Brisbane QLD

**Keywords:** Diabetes complications, Foot diseases, Wound healing, Home care services, hospital‐based, Amputation

## Abstract

**Objectives:**

To assess the incidence, risk factors, and length of stay for hospitalisations, with and without amputations, of people with diabetes‐related foot ulcers (DFU).

**Study design:**

Prospective observational cohort study; secondary analysis of linked Diabetic Foot Services and Queensland Hospital Admitted Patient Data Collection data.

**Settings, participants:**

All people with DFU who visited any of 65 outpatient Diabetic Foot Service clinics in Queensland for the first time during 1 July 2011 – 31 December 2017, followed until first DFU‐related hospitalisation, ulcer healing, or death, censored at 24 months.

**Main outcome measures:**

First overnight hospitalisations for which the principal diagnosis was DFU‐related (International Statistical Classification of Diseases, tenth revision, Australian modification; Australian Classification of Health Interventions codes), by amputation procedure type (none, minor [distal to ankle], major [proximal to ankle]).

**Results:**

Among 4709 people with DFU (median age, 63 years (interquartile range [IQR], 54–72 years); 3275 men [69.5%]; type 2 diabetes, 4284 [91.0%]), DFU‐related hospitalisations were recorded for 977 people (20.7%): 669 without amputations (68.5%), 258 with minor amputations (26.4%), and 50 with major amputations (5.1%). The incidence of first DFU‐related hospitalisations was 50.8 (95% confidence interval [CI], 47.7–54.1) per 100 person‐years lived with DFU before healing, death, or loss to follow‐up. The incidence of first DFU‐related hospitalisation with no amputation was 39.0 (95% CI, 36.2–42.1), with minor amputation 18.0 (95% CI, 17.0–20.0), and with major amputation 5.3 (95% CI, 4.4–6.3) per 100 person‐years with DFU. The median length of stay for DFU‐related hospitalisations was six (IQR, 3–12) days with no amputations, ten (IQR, 5–19) days with minor amputations, and 19 (IQR, 11–38) days with major amputations. The risks of all DFU‐related hospitalisation outcomes were higher for people with deep ulcers or severe peripheral artery disease. The risks of DFU‐related hospitalisation with no amputations were also greater for people aged 37–59 years than for those aged 60 years, and for people with cardiovascular disease, infections, or previous amputations; with minor amputations for people who smoked, had end‐stage renal disease, previous amputations, moderate to severe infections, or peripheral artery disease, or who were not receiving knee‐high offloading or DFU debridement treatments; and with major amputations for people with end‐stage renal disease, peripheral artery disease, or larger ulcers.

**Conclusions:**

The incidence of DFU‐related hospitalisations among people with DFU was high, and most did not involve amputations. Risk factor profiles differed between hospitalisations with or without amputation procedures. Our findings could assist services determine which people with DFU would benefit most from intensive interventions, potentially averting large numbers of diabetes‐related hospitalisations.

**The known**: The diabetes‐related hospitalisation rate is relatively high in Australia, particularly hospitalisations related to diabetes‐related foot ulcers (DFU).

**The new**: The incidence of DFU‐related hospitalisations in the two years following first visits to Diabetic Foot Services is high; 69% of hospitalisations do not involve amputations. Risk factor profiles differed markedly between hospitalisations with or without amputation procedures, including for factors such as age, cardiovascular disease, end‐stage renal disease, infection, peripheral artery disease, ulcer size, and DFU treatments. Median hospital stays range from six days (no amputation) to nineteen days (major amputation).

**The implications**: Evidence‐based profiling of people with DFU could identify who is most likely to benefit from interventions for reducing their risk of hospitalisation.

Diabetes‐related foot ulcers (DFUs) are the leading cause of diabetes‐related hospitalisations and of all‐cause amputations.^1^, ^2^, ^3^ Worldwide, an estimated 18.6 million people lived with DFUs in 2016,^3^ causing about 8.7 million hospitalisations each year, including 1.6 million with amputation procedures.^2^ In Australia, an estimated 50 000 people lived with DFUs in 2017,^4^, ^5^ causing about 28 000 hospitalisations each year, including 5000 with amputation procedures.^4^, ^5^

The diabetes‐related hospitalisation rate for Australia in 2020 (157 per 100 000 resident population) was much higher than the mean for developed nations (102 per 100 000 residents), but the major diabetes‐related amputation rate (proximal to ankle) was much lower in Australia (4.1 *v* 8.5 per 100 000 residents).^2^, ^6^, ^7^, ^8^ However, little is known about risk factors for the DFU‐related hospitalisations that substantially contribute to the high diabetes‐related hospitalisation rate in Australia.^6^

Many studies have reported the incidence of^2^, ^9^, ^10^and risk factors for amputations for people with diabetes,^1^, ^11^, ^12^ but few have reported the incidence of^2^, ^13^, ^14^ and risk factors for DFU‐related hospitalisations of people with diabetes,^14^, ^15^, ^16^ and none for people with DFU. Further, most DFU‐related hospitalisations do not involve amputations, but the reported mean lengths of hospital stays are long (6 to 11 days).^2^, ^13^, ^15^ Investigation of the incidence and risk factors for DFU‐related hospitalisations in people with DFU have consequently been recommended, particularly for comparing hospital admissions with and without amputation procedures.^2^, ^13^, ^15^

We therefore assessed the incidence, risk factors, and length of stay for DFU‐related hospitalisations, with and without amputation, in a large cohort of people with DFU in Queensland.

## Methods

We undertook a secondary analysis of data from a prospective observational cohort study of people with DFU who visited outpatient Diabetic Foot Service clinics in Queensland during July 2011 – December 2017.^17^, ^18^ We report our study according to the Strengthening the Reporting of Observational Studies in Epidemiology statement.^19^

### Study group and settings

We included data for all people with DFU who visited any of 65 outpatient Diabetic Foot Service clinics in Queensland for the first time during 1 July 2011 – 31 December 2017. Participants who attended only once and did not return were excluded. DFU was defined as a break in the skin involving at least part of the dermis below the ankle in a person with diabetes, usually accompanied by peripheral neuropathy or peripheral artery disease in the lower extremity.^20^ The Diabetic Foot Services are located in the sixteen Hospital and Health Service regions in Queensland, except for the Children's Health Queensland Hospital and Health Service, which provides statewide specialist services for children. Sites ranged from small centres in remote towns to large hospitals in major cities. The study cohort included about half the estimated 9000 people with DFU in Queensland, and is one of the largest prospective DFU cohorts worldwide.^17^, ^18^

### Variables

Baseline patient data for 34 demographic, comorbidity, limb, ulcer, and treatment‐related variables were collected during first outpatient Diabetic Foot Service visits (or second visits when data for a variable were not collected during the first visit) (Supporting Information, table 1).^17^, ^18^ All clinical examination and patient‐reported data were collected by trained health professionals using the validated Queensland High Risk Foot Form.^18^, ^21^ For participants with multiple DFUs, the most severe classification for each variable was used, and a combined ulcer size was calculated from all DFUs; the number of DFUs was not recorded.

### Outcomes

The three primary outcomes were the first DFU‐related hospitalisation events, after the first outpatient Diabetic Foot Service visit, with no amputation, minor amputation, or major amputation procedure.

Diabetic Foot Service data were linked with the Queensland Hospital Admitted Patient Data Collection, which captures all Queensland public and private hospital inpatient activities. DFU‐related hospitalisations were defined as overnight hospital admissions for which the principal diagnosis code was a DFU or diabetes‐related foot infection diagnosis code in the International Statistical Classification of Diseases and Related Health Problems, tenth revision, Australian modification (ICD‐10‐AM) or a lower extremity amputation procedure code in the Australian Classification of Health Interventions (Supporting Information, table 1). The DFU‐related hospitalisation outcomes were defined as no amputation (no recorded amputation procedure code), minor amputation (distal to ankle amputation procedure code), or major amputation (proximal to ankle amputation procedure code). If minor and major amputation codes were recorded for a hospitalisation, it was defined as a major amputation hospitalisation.^13^, ^20^

People were followed until the first DFU‐related hospitalisation outcome event for each amputation procedure type, or for a maximum of 24 months. Data for an individual were censored early if they healed (complete epithelialisation of all DFUs, on both feet if applicable) without amputation, died, or were lost to follow‐up (failed to attend a scheduled outpatient visit and were not re‐scheduled), as defined in the validated Queensland High Risk Foot Form.^18^, ^21^ The time to event for the first outcome was defined as the time from the first Diabetic Foot Service visit to the admission date for the hospitalisation outcome event; the length of hospital stay was defined as the time from admission to discharge. If a patient was transferred between hospitals, the combined length of stay was calculated.

### Statistical analyses

The incidence rate (with 95% confidence interval, 95% CI) was calculated for each outcome by dividing their number by the number of person‐years of follow‐up. We calculated medians with interquartile ranges (IQR) for time to event (also depicted in Kaplan–Meier curves) and length of hospital stay; the statistical significance of between‐group differences was assessed in Kruskal–Wallis tests, and that of temporal trend using the Jonckheere–Terpstra method. Risk factors for each outcome were assessed separately using the same procedures. First, associations of variables and the outcome were assessed at the univariable level (univariable Cox proportional hazard regression). Second, a multivariable flexible parametric survival model was built by entering all variables for which *P* < 0.1 in the univariable analysis and using model specifications based on the Akaike information criterion (AIC) and the Bayesian information criterion (BIC) (Supporting Information, table 2). Third, age was included as a continuous variable and transformed using restricted cubic splines (degrees of freedom based on lowest AIC and BIC); we report the adjusted hazard ratio (aHR) for each year of age. Fourth, model fit was checked by examining martingale residuals. When the missing data proportion was smaller than 10%, the variable was included in analyses and the cases with missing data were omitted; when the proportion was 10–25%, a missing data category was added for the variable; when the proportion exceeded 25%, the variable was excluded from analyses. All analyses were performed in Stata/SE 16.1, and the user‐written Stata package *stpm2* and *stpm2_standsurv* were used for flexible parametric modelling.^22^

### Ethics approval

The study was approved by the human research ethics committees of the Prince Charles Hospital (HREC/15/QPCH/155) and the Queensland University of Technology (1800000722); a *Public Health Act 2005* waiver was approved by the Queensland Health Office of Research and Innovation (RD007685) to use deidentified data without individual consent for the study.

## Results

Of 4832 eligible participants, 123 (2.5%) did not return for second Diabetic Foot Service visits and were excluded from the study (Box 1). The median age of the 4709 people included was 63 years (IQR, 54–72 years); 3275 were men (69.5%), 4284 had type 2 diabetes (91.0%), 2486 lived in major cities (54.6%), and 495 were Indigenous Australians (10.5%) (Box 2). During follow‐up of 1914 person‐years (median, 2.3 [IQR, 0.9–6.0] months per person), first DFU‐related hospitalisation outcomes were recorded for 977 people (20.7%): no amputation procedure, 669 (68.5%); minor amputation procedure, 258 (26.4%), and major amputation procedure, 50 (5.1%) (Box 1; Supporting Information, figure 1). A total of 2875 people had healed (61.1%), 430 were lost to follow‐up (9.1%), and 211 had died (4.5%); 216 people (4.6%) were alive, unhealed, and had not been hospitalised by two years after their first clinic visit (Box 1).

Box 1 **Selection and outcomes for people with diabetes‐related foot ulcers who visited Queensland Diabetic Foot Service clinics for the first time during 1 July 2011 – 31 December 2017**

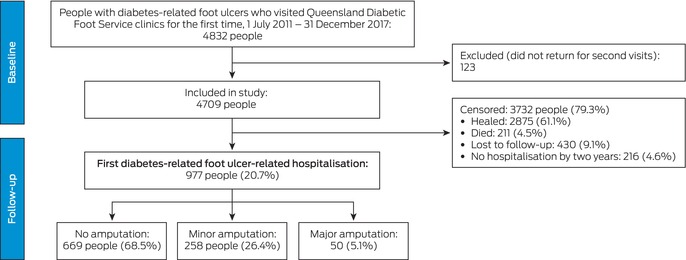

Box 2 **Baseline characteristics of people with diabetes‐related foot ulcers (DFU) who visited Queensland Diabetic Foot Service clinics for the first time during 1 July 2011 – 31 December 2017, and unadjusted incidence rates for first DFU‐related hospitalisation outcomes before healing, death, or loss to follow‐up (censored at 24 months)**

**Table:** 

Characteristics	Number of people with data	Number of people	Incidence rate, per 100 person‐years (95% CI)		
			No amputation	Minor amputation	Major amputation
**All people**	4709	4709	39.0 (36.2–42.1)	18.0 (17.0–20.0)	5.3 (4.4–6.3)
**Demographic characteristics**					
Sex	4709				
Male		3275 (69.5%)	38.8 (35.4–42.5)	19.4 (17.3–21.7)	5.3 (4.3–6.5)
Female		1434 (30.5%)	39.6 (34.6–45.3)	16.9 (14.0–20.3)	5.3 (3.8–7.3)
Age (years), median (IQR)	4708	63 (54–72)	—	—	—
Indigenous status	4709				
Aboriginal or Torres Strait Islander		495 (10.5%)	54.9 (45.6–66.1)	20.9 (16.1–27.1)	7.2 (4.7–11)
Non‐Indigenous		4214 (89.5%)	37.0 (34.1–40.1)	18.3 (16.5–20.3)	5.0 (4.1–6.1)
Geographic remoteness^23^	4559				
Major city		2486 (54.6%)	40.3 (36.0–45.1)	18.2 (15.7–21.1)	4.9 (3.7–6.5)
Inner/outer regional area		1857 (40.7%)	36.5 (32.6–40.9)	18.6 (16.1–21.5)	5.7 (4.5–7.3)
Remote/very remote area		216 (4.7%)	52.7 (40.4–68.6)	22.3 (15.6–31.9)	2.1 (0.7–6.5)
**Medical conditions**					
Diabetes	4709				
Type 1		425 (9.0%)	45.8 (36.4–57.7)	19.1 (13.9–26.2)	4.7 (2.5–8.7)
Type 2		4284 (91.0%)	38.4 (35.4–41.5)	18.6 (16.8–20.6)	5.3 (4.4–6.4)
Duration (years), median (IQR)	1736	15 (8–22)	—	—	—
HbA_1c_ (mmol/mol), median (IQR)	1203	64 (52–82)	—	—	—
Hypertension	4709				
Yes		2502 (53.1%)	40.8 (36.7–45.4)	19.3 (16.8–22.1)	5.4 (4.2–6.9)
No		2207 (46.9%)	37.4 (33.6–41.6)	18.1 (15.8–20.7)	5.2 (4.0–6.6)
Dyslipidaemia	4709				
Yes		1701 (36.1%)	40.2 (35.6–45.4)	18.6 (15.8–21.8)	5.5 (4.2–7.4)
No		3008 (63.9%)	38.3 (34.8–42.2)	18.6 (16.5–21.1)	5.1 (4.1–6.4)
Cardiovascular disease	4709				
Yes		986 (20.9%)	45.7 (39.1–53.3)	17.5 (14.0–21.8)	7.1 (5.1–10)
No		3723 (79.1%)	37.4 (34.3–40.7)	18.9 (17.0–21.1)	4.8 (3.9–5.9)
Chronic kidney disease	4709				
Yes		620 (13.2%)	43.4 (35.6–52.9)	18.5 (14.1–24.2)	6.4 (4.1–10)
No		4089 (86.8%)	38.4 (35.4–41.6)	18.6 (16.8–20.7)	5.1 (4.2–6.2)
End‐stage renal failure	4709				
Yes		185 (3.9%)	51.5 (36.8–72.1)	37.6 (26.6–53.2)	19 (12–30)
No		4524 (96.1%)	38.5 (35.7–41.6)	17.9 (16.1–19.8)	4.7 (3.9–5.7)
Smokes	4709				
Yes		494 (10.5%)	40.5 (32.8–49.8)	20.0 (15.5–25.9)	6.6 (4.3–10)
No		4215 (89.5%)	38.8 (35.8–42.1)	18.4 (16.6–20.5)	5.1 (4.2–6.2)
**Limb**					
Previous foot ulcer	4709				
Yes		3621 (76.9%)	40.0 (36.8–43.6)	18.4 (16.5–20.6)	5.6 (4.6–6.8)
No		1088 (23.1%)	35.8 (30.4–42.1)	19.4 (15.9–23.8)	4.2 (2.8–6.4)
Previous amputation	4697				
Yes		1428 (30.4%)	47.2 (41.9–53.2)	24.8 (21.5–28.6)	7.9 (6.2–10.1)
No		3281 (69.6%)	35.2 (31.9–38.7)	15.4 (13.5–17.6)	3.9 (3.0–5.0)
Neuropathy	3866				
Yes		3319 (85.9%)	37.9 (34.7–41.4)	19.2 (17.2–21.5)	5.4 (4.4–6.6)
No		547 (14.1%)	36.0 (28.2–46.0)	13.6 (9.5–19.4)	2.6 (1.2–5.8)
Peripheral artery disease	3800				
No		2214 (58.3%)	33.4 (29.7–37.6)	15.4 (13.2–18.1)	2.7 (1.9–3.9)
Mild to moderate		1357 (35.7%)	38.1 (33.4–43.5)	19.5 (16.5–23.0)	6.6 (5.0–8.7)
Severe		229 (6.0%)	68.9 (52.5–90.5)	38.0 (28.3–51.1)	17 (11–26)
Foot deformity	3039				
Yes		1910 (62.8%)	40.6 (36.4–45.3)	18.6 (16.1–21.5)	6.4 (5.1–8.1)
No		1129 (37.2%)	38.7 (33.2–45.1)	16.3 (13.2–20.1)	3.9 (2.6–5.9)
Acute Charcot foot	3768				
Yes		69 (1.8%)	29.9 (15.6–57.5)	14.4 (6.5–32.1)	2.3 (0.3–16)
No		3699 (98.2%)	38.5 (35.5–41.9)	18.4 (16.5–20.5)	5.1 (4.2–6.3)
**Ulcer**					
Ulcer size (cm^2^), median (IQR)	3597	0.70 (0.16–2.38)	—	—	—
Ulcer size					
Small (< 1 cm^2^)		2038 (56.7%)	30.8 (26.8–35.3)	13.0 (10.7–15.7)	2.5 (1.6–3.8)
Medium (1–3 cm^2^)		818 (22.7%)	39.1 (33.0–46.3)	23.7 (19.4–28.9)	5.4 (3.7–8.1)
Large (> 3 cm^2^)		741 (20.6%)	52.3 (45.0–60.8)	21.6 (17.7–26.4)	8.5 (6.3–12)
Deep ulcer	4654				
Yes		728 (15.6%)	62.1 (53.3–72.3)	36.2 (30.7–42.7)	9.6 (7.1–13)
No		3926 (84.4%)	35.0 (32.1–38.1)	14.7 (13.0–16.6)	4.3 (3.5–5.4)
Infection	4702				
No		3106 (66.1%)	32.9 (29.8–36.5)	14.0 (12.2–16.1)	4.5 (3.5–5.7)
Mild		994 (21.1%)	41.4 (35.6–48.1)	21.5 (17.8–25.9)	6.3 (4.5–8.7)
Moderate to severe		602 (12.8%)	69.7 (58.9–82.4)	37.2 (30.7–45.1)	7.4 (4.9–11)
**Recent diabetes‐related foot ulcer treatment provider**					
Podiatrist	4709				
Yes		4491 (95.4%)	38.3 (35.5–41.4)	18.5 (16.7–20.4)	5.4 (4.5–6.5)
No		218 (4.6%)	51.9 (39.0–69.1)	21.3 (14.6–31.0)	2.9 (1.1–7.8)
General practitioner	4709				
Yes		420 (8.9%)	36.8 (28.1–48.3)	24.2 (17.9–32.7)	5.4 (2.9–10)
No		4289 (91.1%)	39.2 (36.3–42.4)	18.2 (16.4–20.1)	5.3 (4.4–6.3)
Surgical specialist	4709				
Yes		266 (5.6%)	60.1 (46.9–76.9)	35.4 (26.8–46.9)	7.8 (4.4–14)
No		4443 (94.4%)	37.7 (34.8–40.8)	17.5 (15.8–19.4)	5.1 (4.2–6.1)
Medical specialist	4709				
Yes		557 (11.8%)	44.1 (36.1–53.8)	25.5 (20.3–32.1)	4.8 (2.9–8.0)
No		4152 (88.2%)	38.3 (35.3–41.6)	17.6 (15.8–19.6)	5.3 (4.4–6.5)
Nurse	4709				
Yes		1124 (23.9%)	42.7 (37.0–49.3)	23.4 (19.7–27.8)	6.0 (4.4–8.4)
No		3585 (76.1%)	37.8 (34.6–41.3)	17.0 (15.1–19.1)	5.0 (4.1–6.2)
Other	4709				
Yes		565 (12.0%)	49.5 (40.3–60.8)	20.7 (15.7–27.3)	5.1 (2.9–8.7)
No		4144 (88.0%)	37.8 (34.9–41.0)	18.4 (16.6–20.4)	5.3 (4.4–6.4)
**Current diabetes‐related foot ulcer treatment type**					
Debrided ulcer	3772				
Yes		3357 (89.0%)	39.4 (36.1–43.0)	17.1 (15.2–19.1)	5.3 (4.3–6.5)
No		415 (11.0%)	53.6 (42.1–68.2)	29.0 (21.7–38.9)	6.5 (3.6–12)
Dressing appropriate	3772				
Yes		3644 (96.6%)	40.9 (37.7–44.4)	18.0 (16.2–20.1)	5.5 (4.6–6.7)
No		128 (3.4%)	44.1 (26.6–73.1)	23.9 (12.9–44.5)	0
Antibiotics prescribed	4699				
Yes		1697 (36.1%)	53.3 (47.6–59.7)	28.7 (25.1–32.8)	7.0 (5.4–9.1)
No		3002 (63.9%)	32.5 (29.3–36.0)	13.5 (11.7–15.6)	4.3 (3.3–5.5)
Knee‐high offloading	4706				
Yes		1835 (39.0%)	37.8 (33.6–42.6)	15.7 (13.3–18.5)	6.0 (4.6–7.8)
No		2871 (61%)	41.9 (37.6–46.8)	21.4 (18.7–24.5)	4.3 (3.2–5.7)
Footwear appropriate	4686				
Yes		2743 (58.5%)	36.2 (32.6–40.1)	18.4 (16.1–20.9)	5.5 (4.4–7.0)
No		1943 (41.5%)	42.8 (38.1–48.1)	18.6 (16.0–21.7)	4.7 (3.5–6.3)
Patient education about foot‐related self‐care provided	3772				
Yes		3721 (98.6%)	40.7 (37.5–44.2)	18.3 (16.4–20.3)	5.5 (4.6–6.7)
No		51 (1.4%)	46.6 (20.9–104)	11.6 (2.9–46.3)	5.4 (0.8–38)

CI = confidence interval; Hb_A1c_ = glycated haemoglobin; IQR = interquartile range.

### Incidence of diabetes‐related foot ulcer‐related hospitalisation

Among people with DFU, the incidence rate of first DFU‐related hospitalisation was 50.8 (95% CI, 47.7–54.1) per 100 person‐years lived with DFU before healing, death, or loss to follow‐up. The incidence of first DFU‐related hospitalisation with no amputation procedure was 39.0 (95% CI, 36.2–42.1) per 100 person‐years with DFU, with minor amputation procedure was 18.0 (95% CI, 17.0–20.0) per 100 person‐years with DFU, and with major amputation procedure was 5.3 (95% CI, 4.4–6.3) per 100 person‐years with DFU (Box 2).

The median time to first hospitalisation was 85 (IQR, 32–238) days with no amputation procedure, 77 (IQR, 23–195) days with minor amputation procedures (*v* no amputation: *P* = 0.07), and 96 (IQR, 29–271) days with major amputation procedures (*v* no amputation, *P* = 0.039; *v* minor amputation, *P* = 0.021). The median length of stay for first hospitalisation was six (IQR, 3–12) days with no amputation, ten (IQR, 5–19) days with minor amputation procedures, and 19 (IQR, 11–38) days with major amputation procedures (trend: *P* < 0.001).

### Risk factors for diabetes‐related foot ulcer‐related hospitalisation

The adjusted risk of hospitalisation with no amputation was higher for people with cardiovascular disease (aHR, 1.36; 95% CI, 1.09–1.69), previous amputation (aHR, 1.39; 95% CI, 1.13–1.71), severe peripheral artery disease (aHR, 1.77; 95% CI, 1.19–2.64), any infection severity (mild: aHR, 1.29; 95% CI, 1.02–1.63; moderate to severe: aHR, 1.47; 95% CI, 1.10–1.96), or deep ulcers (aHR; 95% CI, 1.28; 1.02–1.61) (Box 3). Compared with people aged 60 years, the risk of hospitalisation without amputation was higher for people aged 37–59 years (37 years: aHR, 1.32; 95% CI, 1.007–1.73; 59 years: aHR, 1.02; 95% CI, 1.002–1.03), and lower for people aged 61–64 years (61 years: aHR, 0.98; 95% CI: 0.97–0.999; 64 years: aHR, 0.94; 95% CI, 0.88–0.999) (Box 4).

Box 3 Independent associations between baseline characteristics of people with diabetes‐related foot ulcers (DFU) who visited Queensland Diabetic Foot Service clinics for the first time during 1 July 2011 – 31 December 2017, and DFU‐related hospitalisation outcomes: multivariable flexible parametric survival analyses (censored at 24 months)*

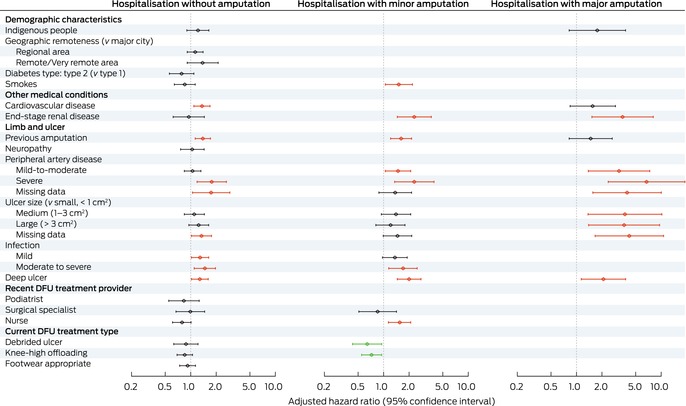

* The data underlying this figure are included in the Supporting Information, table 3. The analyses by outcome category (no amputation, minor amputation, major amputation) include variables for which *P* < 0.1 in univariable analyses (Supporting Information, table 4); excluded variables are not included in this figure. Age was included as a continuous variable; results by age are included in Box 4. Red: increased risk; green: reduced risk; grey: no statistically significant difference in risk. The variables neuropathy, peripheral artery disease, ulcer size, and debrided ulcer included a missing data category (19–25% missing values).

Box 4 Independent associations between the age of people with diabetes‐related foot ulcers (DFU) who visited Queensland Diabetic Foot Service clinics for the first time during 1 July 2011 – 31 December 2017, and first DFU‐related hospitalisation outcomes (reference: 60 years of age): multivariable flexible parametric survival analyses (censored at 24 months)*

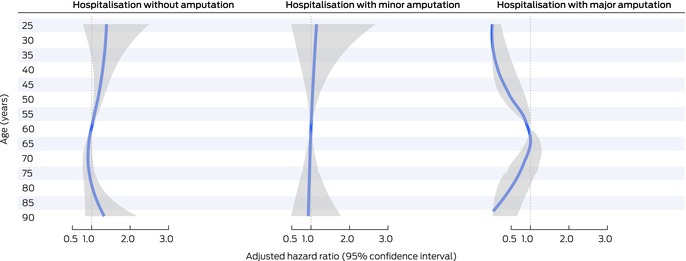

* Each analysis is adjusted for the corresponding variables in Box 3. The adjusted hazard ratios for all ages are depicted as a blue line, the 95% confidence interval as a grey band. The data for this graph are included in the Supporting Information, table 5; the adjusted hazard ratios for 5‐year age points are included in the Supporting Information, figure 2.

The risk of hospitalisation with a minor amputation procedure was higher for people with end‐stage renal disease (aHR, 2.30; 95% CI, 1.45–3.66), previous amputation (aHR, 1.61; 95% CI, 1.21–2.14), peripheral artery disease (severe: aHR, 2.30; 95% CI, 1.35–3.94; mild to moderate: aHR, 1.48; 95% CI, 1.05–2.09), moderate to severe infection (aHR, 1.70; 95% CI, 1.15–2.49), deep ulcers (aHR, 2.00; 95% CI, 1.45–2.76), or who smoked (aHR, 1.51; 95% CI, 1.05–2.19) or received DFU treatment from a nurse (aHR, 1.55; 95% CI, 1.14–2.09); the risk was lower for people who received DFU debridement (aHR, 0.64; 95% CI, 0.43–0.95) or knee‐high offloading treatment (aHR, 0.72; 95% CI, 0.55–0.95) (Box 3). The risk of hospitalisation with a minor amputation procedure did not differ by age from that for people aged 60 years (Box 4).

The risk of hospitalisation with a major amputation procedure was higher for people with end‐stage renal disease (aHR, 3.50; 95% CI, 1.52–8.07), peripheral artery disease (severe: aHR, 6.72; 95% CI, 2.37–19.0; mild to moderate: aHR, 3.18; 95% CI, 1.38–7.32), larger ulcers (*v* small: medium: aHR, 3.73; 95% CI, 1.37–10.2; large: aHR, 3.65; 95% CI, 1.39–9.58), or deep ulcers (aHR, 2.08; 95% CI, 1.14–3.80) (Box 3). Compared with people aged 60 years, the risk of hospitalisation with a major amputation procedure was lower for people aged 56 years or younger and for people aged 80 years or older (56 years: aHR, 0.82; 95% CI, 0.67–0.99; 80 years: aHR, 0.38; 95% CI, 0.15–0.98) (Box 4).

## Discussion

In our large cohort of people with DFU followed during 2011–19, the incidence of first DFU‐related hospital admissions was high (51 per 100 person‐years lived with DFU before healing, death, or lost to follow‐up); 68.5% of these hospitalisations did not involve amputation procedures. The median time to first hospitalisation was about three months for people in all three outcome categories, but the median length of hospital stay ranged from six days when no amputation was involved to 19 days for admissions involving major amputations. Two risk factors were common to all hospitalisation outcomes (deep ulcers and severe peripheral artery disease), but others differed by outcome: for example, the risks of hospitalisation without amputation were greater for people aged 37–59 years than for those aged 60 years and for people with cardiovascular disease; with minor amputations for people who smoked or were not receiving knee‐high offloading treatments; and with major amputations for people with larger ulcers.

Our finding that DFU‐related hospitalisations were recorded for 20.7% of people during a maximum 24‐month follow‐up is consistent with European findings of 21–62% of people with DFU during 6–24 months of follow‐up.^24^, ^25^, ^26^ Further, the baseline characteristics of our cohort were similar to those of a recently described large outpatient cohort of people with DFU in the United Kingdom, particularly with respect to peripheral artery disease (Queensland, 42%; United Kingdom, 35%), infection (Queensland, 34%; United Kingdom, 40%), and deep ulcers (Queensland, 16%; United Kingdom, 17%).^27^ Given these similarities with other large prospective studies, our findings can probably be generalised to people with DFU attending diabetic foot services in other parts of the world. Further, as our cohort included almost all people with DFU attending Queensland Diabetic Foot Services and about half of all people in Queensland with DFU,^17^, ^18^ and Australian guidelines recommend that all people with DFU attend Diabetic Foot Services,^4^ people not included in our study were probably managed by primary health care professionals who did not consider the DFU severe enough for referral to Diabetic Foot Services. Our cohort is probably representative of people with DFU who attend Diabetic Foot Services, but the severity of their condition may be greater than for all people with DFU.

We found that about half of all first DFU‐related hospitalisations were within three months of first outpatient Diabetic Foot Service visits; median hospital stays ranged from six days with no amputation to ten days with minor amputation and 19 days with major amputation. As the mean hospital length of stay for diabetes‐related hospitalisations is five to seven days in England^28^ and the United States,^29^ and four to six days for all‐cause hospitalisations in Australia,^30^ these DFU‐related hospital stays were all relatively long, regardless of amputation type. The first three months after the first visit to an outpatient Diabetic Foot Service seem to be critical for averting long DFU‐related hospitalisations of people with diabetes, who are at very high risk of hospitalisation.

We found that some risk factors for hospitalisations without amputation were common to all DFU‐related hospitalisations, such as severe peripheral artery disease and deep ulcers, but others were significant only for hospitalisations without amputation procedures, such as cardiovascular disease, mild infection, and younger age (37–59 years, compared with people aged 60 years or older). Other studies of people with diabetes have also reported that cardiovascular disease was a risk factor for DFU‐related hospitalisation without amputation, and the authors of these studies suggested that the admissions were from services with less expertise in managing DFU.^15^, ^16^ Further, although mild infection has not been specifically reported as a risk factor, DFU guidelines recommend that people with mild infections be managed as outpatients.^4^, ^31^ Our finding that people under 60 years of age are at greater risk of hospitalisations without amputation than people aged 60 years or older is consistent with recent reports that younger people are at greater risk than older people of other poor DFU outcomes, such as non‐healing and infection,^1^, ^32^, ^33^ and that DFU‐related hospitalisation rates have increased most rapidly in younger age groups.^13^, ^14^, ^16^, ^34^, ^35^ Younger people with DFU may have younger onset type 2 diabetes, a more aggressive phenotype, particularly with regard to neuropathy,^1^, ^13^, ^14^, ^18^, ^32^ and they are more active than older people, increasing plantar tissue stress that can lead to poorer DFU outcomes.^1^, ^14^, ^18^, ^32^ Greater access to intensive cardiovascular disease management and specialist outpatient Diabetic Foot Services could be useful strategies for averting DFU‐related hospitalisations, especially among younger people.^1^, ^2^, ^31^, ^36^, ^37^

Age did not influence the risk of hospitalisations with minor amputations, but the risk of major amputation was lower for people under 57 years of age or aged 80 years or older than for people aged 60 years. This suggests that age is a critical factor when making decisions about major amputations for people hospitalised for DFU.^38^, ^39^, ^40^ This may be because surgeons and patients prefer attempting limb‐sparing treatments, such as revascularisation procedures, to prevent or delay major amputations and their effects on function in younger people and the risk of death in older patients.^38^, ^39^, ^40^ Decision making regarding amputations for people hospitalised for DFU should be further investigated.

Peripheral artery disease and infection also influenced the risk of DFU‐related hospitalisation outcomes. Both are recognised risk factors for amputation,^1^, ^11^ but our findings indicate that their severity influences their impact. Any infection (mild to severe) was a risk factor for hospitalisation without amputation, moderate to severe infection was a risk factor for hospitalisation with minor amputation, but infection was not a risk factor for major amputations. Conversely, any peripheral artery disease (mild to severe) was a risk factor for hospitalisations with minor and major amputations — the increase in risk of major amputation associated with severe peripheral artery disease was greater than for any other risk factor — but mild to moderate peripheral artery disease was not a risk factor for hospitalisation without amputation. However, we collected information on these factors at first Diabetic Foot Service visits, and subsequent changes in status could have affected our findings. Whether changes in risk factor status over time influences DFU‐related hospitalisation risk should be investigated.

Other risk factors for amputation we identified have been reported in other studies, including end‐stage renal disease, smoking, previous amputation, and larger ulcer sizes.^1^, ^11^, ^12^ The risk factors for minor amputations were mostly modifiable (such as smoking and not receiving DFU treatments); for major amputations they were mostly non‐modifiable (such as larger ulcer sizes), but could perhaps be modified if people were referred to outpatient Diabetic Foot Services that adhere to guideline‐recommended treatments earlier.^1^, ^2^, ^31^, ^36^

### Limitations

Information for some variables was based on reports by the people attending the outpatient clinics, but all data were recorded by trained clinicians using a validated tool.^17^, ^18^, ^21^, ^32^ To minimise bias caused by missing data, we excluded diabetes duration and glycated haemoglobin as factors from our analyses, which may have affected our findings. As we collected data at baseline, our findings primarily concern the prognostic value of patient status at their first visit to outpatient services, although a very small number of participants could have visited these services prior to July 2011 (only limited services were available before this time), and data for some variables may have changed over time. As we relied on ICD‐10‐AM coding to identify hospitalisation outcomes we may have missed some admissions, such as hospitalisations with sepsis caused by DFU. However, the number missed was probably small, as the accuracy of the ICD‐10‐AM codes for identifying DFU‐related hospitalisations is high.^2^, ^13^, ^20^ We reported overall length of hospital stay, as is usual in DFU‐related articles, but not by inpatient subtype (such as acute or rehabilitation), which could be important for policy makers. We examined the first DFU‐related hospitalisation before healing after the first outpatient visit, but risk factors for subsequent DFU‐related and all‐cause hospitalisations could be different. We considered the first outpatient visit factors and first DFU‐related hospitalisation outcomes to be of greatest importance for clinicians in Diabetic Foot Services. We used the validated Queensland High Risk Foot Form tool to capture deaths, and may have missed some censored outcomes, but the mortality rate was similar in another large cohort of people with DFU with a similar follow‐up period (twelve weeks: Queensland, 4.5%; United Kingdom, 4.2%).^27^ Finally, our findings underestimate the incidence of all DFU‐related hospitalisations and all‐cause hospitalisations, as we included only hospitalisations that were the first DFU‐related hospitalisations and those that were primarily DFU‐related, but we also censored the 4.6% of our cohort who remained unhealed two years after their first clinic visit, which would increase our estimate.

### Conclusion

Among people with DFU who have visited a Queensland Diabetic Foot Service for the first time, the incidence of DFU‐related hospitalisations before they heal is very high, but most do not require amputations. Risk factor profiles differed between hospitalisations with or without amputation procedures, including for factors such as age, cardiovascular disease, end‐stage renal disease, infection, peripheral artery disease, ulcer size, and DFU treatments. Our findings regarding the comparatively large number of hospitalisations of people with DFU could assist services determine who would benefit most from intensive interventions and potentially avert large numbers of diabetes‐related hospitalisations in Australia and overseas.

## Open access

Open access publishing facilitated by Queensland University of Technology, as part of the Wiley – Queensland University of Technology agreement via the Council of Australian University Librarians.

## Competing interests

Peter A Lazzarini and Jaap J van Netten are members respectively of the International Working Group on the Diabetic Foot (IWGDF) working groups and editorial board, which are responsible for authoring international evidence‐based guidelines on diabetes foot disease management. Peter A Lazzarini was also co‐chair of Diabetes Feet Australia and the Australian evidence‐based guidelines for the prevention and management of diabetes‐related foot disease. The authors declare that there are no other relationships or activities that might bias, or be perceived to bias, their work.

## Data sharing

The data that support the findings of this study are not publicly available but will be shared after approval by the relevant ethics committees and data custodians: the Queensland Statewide Diabetes Clinical Network and the Queensland Health Statistical Services Branch (https://www.health.qld.gov.au/hsu/research).

Received 10 May 2024, accepted 6 January 2025

## Supporting information

Supplementary methods and results
