# Supplementary material for: The incidence of and risk factors for hospitalisations and amputations for people with diabetes‐related foot ulcers in Queensland, 2011–19: an observational cohort study
Source: Med J Aust. 2025 Jul 13;223(3):149–58. doi: 10.5694/mja2.52703 (PMC12318492; doi:10.5694/mja2.52703)
Supplement: Supplementary file 1 — Supplementary methods and results [file MJA2-223-149-s001.pdf]

Supporting Information

Supplementary methods and results

This appendix was part of the submitted manuscript and has been peer reviewed.
It is posted as supplied by the authors.

Appendix to: Zhang Y, Cramb SM, McPhail SM, et al. The incidence and risk factors for hospitalisations and amputations among people with diabetes-related foot ulcers in Queensland, Australia; 2011-19: an observational cohort study. *Med J Aust* 2025; doi: 10.5694/mja2.52703.

Supplementary methods

Table 1. Definitions of included variables

Variables	Definitions
**Variables**	**Definitions**
**Demographics** (from self-report)	
Sex	Participant identifies as male, female or indeterminate/intersex
Age	Participant's age in years at time of the first visit
Indigenous status	Participant identifies as being of Aboriginal and/or Torres Strait Islander origin and is respectfully referred to as being Indigenous for the purpose of the study
Geographic remoteness	Participant's residential postcode was transformed into geographical remoteness areas (major city, regional area (inner or outer regional area), remote area (remote or very remote area)), according to Remoteness Areas Index of Australia (Australian Bureau of Statistics)
**Comorbidity** (from self-report)	
Diabetes type	Participant has been diagnosed with Type1 or Type 2 diabetes mellitus
Diabetes duration (years)	Year participant was diagnosed was used to calculate diabetes duration
Glycated haemoglobin (HbA1c)	The participant's most recent reported glycated haemoglobin level (HbA1c). HbA1c % was converted into mmol/mol.
Hypertension	Participant has been diagnosed with hypertension: Blood pressure of >140mmHg systolic and/or >90mmHg diastolic.
Dyslipidemia	Participant has been diagnosed with dyslipidemia: Low-density lipoprotein cholesterol >2.5 mmol/L, Triglycerides >2.0mmol/L or Cholesterol >6.2mmol/L.
Cardiovascular disease	Participant has been diagnosed with cardiovascular disease: All diseases and conditions of the heart and blood vessels, including myocardial infarction, angina or stroke.
Chronic kidney disease	Participant has been diagnosed with chronic kidney disease: estimated glomerular filtration rate (eGFR) <90mL/min/1.73m^2.
End stage renal disease	Participant has been diagnosed with end stage renal disease: estimated glomerular filtration rate (eGFR) <15 mL/min/1.73m^2, on dialysis and/or had a kidney transplant.
Smoker	Participant smokes tobacco regularly or has smoked in the previous 4 weeks.
**Limb** (from clinical diagnoses)	
Previous foot ulcer	History of a previous healed foot ulcer. Participant self-report is acceptable.
Previous amputation	The participant has had an amputation procedure through (part of) the lower limb confirmed on clinical examination
Neuropathy	Lack of protective sensation to a 10-gram monofilament on at least 2 of 3 plantar forefoot locations
Peripheral artery disease	Mild-to-moderate PAD: Toe systolic pressure 30-70mmHg<br>Severe PAD: Toe systolic pressure <30mmHg
Foot deformity	Scored at least 3 points on a 6-point foot deformity score (one point each scored if small muscle wasting, Charcot foot deformity, bony prominence, prominent metatarsal heads, hammer/claw toes, or limited joint mobility present).
Acute Charcot foot	Suspected Acute Charcot foot due to currently having a red, hot, swollen, unilateral neuropathic foot joint without an ulcer in close proximity.
**Ulcer** (from clinical diagnoses)	
Ulcer size	Ulcer surface area was estimated by multiplying length of ulcer in mm by width of ulcer in mm. Participants with multiple ulcers had the surface area of all ulcers summed together for a combined ulcer surface area in mm^2. Ulcer surface area was then categorised into: small (<1cm^2), medium (1-3cm^2), and large (>3cm^2).
Deep ulcer	Ulcer penetrating to tendon, capsule, bone or joint, including University of Texas Wound Classification system depth categories of 2 or 3
Infection	At least 2 of the following signs or symptoms were present around the ulcer: erythema, swelling, warmth, tenderness or pain, purulent discharge.<br>Mild infection: Erythema extends <2cm from the edge of the ulcer<br>Moderate-to-severe infection: Erythema extends >2cm from the edge of the ulcer +/- systemic signs or symptoms of infection.

**Recent DFU treatment by**: (from self-report)	
Podiatrist	A podiatrist provided treatment for the participant's foot complication in the week prior to, or at, the current visit to a Diabetic Foot Service.
General practitioner (GP)	A GP provided treatment for the participant's foot complication in the week prior to, or at, the current visit to a Diabetic Foot Service.
Surgical specialist	A surgical specialist provided treatment for the participant's foot complication in the week prior to, or at, the current visit to a Diabetic Foot Service.
Medical specialist	A medical specialist physician provided treatment for the participant's foot complication in the week prior to, or at, the current visit to a Diabetic Foot Service.
Nurse	A nurse provided treatment for the participant's foot complication in the week prior to, or at, the current visit to a Diabetic Foot Service.
Others	Other health professionals provided treatment for the participant's foot complication in the week prior to, or at, the current visit to a Diabetic Foot Service.
**Current DFU treatment** (from clinically diagnoses)	
Debrided	Sharp debridement of ulcer performed in the current visit.
Dressing appropriate	Dressing applied during current visit was considered appropriate if it promoted a moist wound healing environment unless clinically contraindicated.
Antibiotics prescribed	Antibiotic therapy commenced in the current visit if needed (in participants with ulcers that are infected or non-healing) or medical practitioner has prescribed or been contacted to prescribe antibiotic therapy
Knee-high offloading	Knee-high offloading device (removable or non-removable) is already used or has been prescribed in the current visit.
Footwear appropriate	Footwear was considered appropriate for the contralateral foot in the current visit if it protects against injury, allowed appropriate offloading if required (such as insoles) and encouraged safe mobility.
Patient educated	Participant was provided education on foot-related self-care in the current visit.
**DFU-caused hospitalisation identified from hospital dataset**	
Hospitalisation with no amputation	ICD-10-AM (the International Statistical Classification of Diseases and Related Health Problems, Tenth Revision, Australian Modification) diagnosis codes: E1073, E1173, E1373, E1473, L0302, L0311, M8617, M8627, M8647, M8667, M8697
Hospitalisation with minor amputation	Australian Classification of Health Interventions (ACHI) procedure codes 4433800, 4435800, 9055700, 4436100, 4436101, 4436400, 4436401
Hospitalisation with major amputation	ACHI procedure codes: 4436701, 4436702, 4437000, 4437300, 4436700

DFU: diabetes-related foot ulcer

Table 2. Model specifications of multivariable flexible parametric survival analysis

	Number of variables included	Model specification	Age (restricted cubic splines)
DFU-caused hospitalisation with no amputation	19	Hazard scale, 2 interior knots and 2 boundary knots	3 degrees of freedom
DFU-caused hospitalisation with minor amputation	12	Hazard scale, 1 interior knot and 2 boundary knots	2 degrees of freedom
DFU-caused hospitalisation with major amputation	8	Hazard scale, 1 interior knot and 2 boundary knots	2 degrees of freedom

DFU: diabetes-related foot ulcer.

Supplementary results

Figure 1. Unadjusted Kaplan-Meier curves in people with diabetes-related foot ulcers for first diabetes-related foot ulcer-caused hospitalisation with no amputation, minor amputation, and major amputation (censored at 24 months)

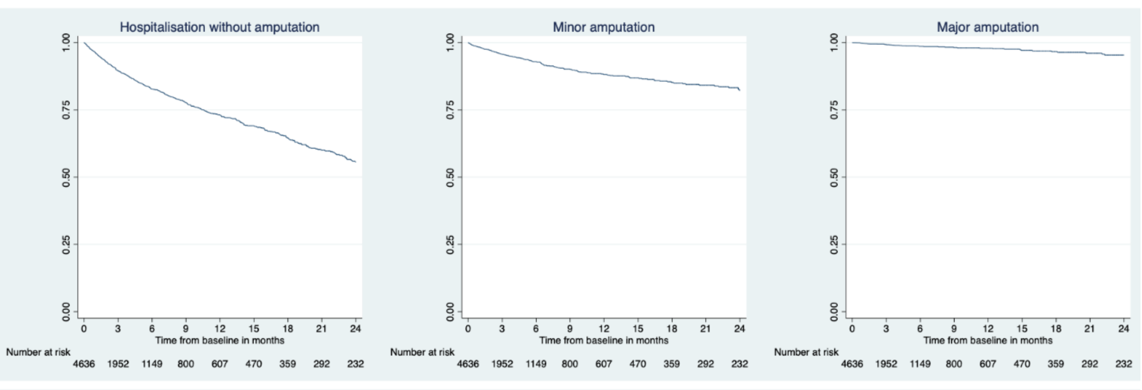

Table 3. Adjusted hazard ratios in people with diabetes-related foot ulcers for first diabetes-related foot ulcer-caused hospitalisation with no amputation, minor amputation, and major amputation: multivariable flexible parametric survival analyses (censored at 24 months)*

	Adjusted hazard ratio (95% confidence interval)		
**Characteristic**	**No amputation**	**Minor amputation**	**Major amputation**
**Demographic characteristics**			
Indigenous people	1.22 (0.90–1.64)	—	1.76 (0.82–3.80)
Geographic remoteness (*v* major city)			
Regional area	1.13 (0.91–1.40)	—	—
Remote/very remote area	1.38 (0.91–2.11)	—	—
Diabetes type: type 2 (*v* type 1)	0.78 (0.56–1.09)	—	—
Smokes	0.85 (0.64–1.13)	1.51 (1.05–2.19)	—
**Other medical conditions**			
Cardiovascular disease	1.36 (1.09–1.69)	—	1.55 (0.84–2.89)
End-stage renal disease	0.95 (0.62–1.45)	2.30 (1.45–3.66)	3.50 (1.52–8.07)
**Limb and ulcer**			
Previous amputation	1.39 (1.13–1.71)	1.61 (1.21–2.14)	1.47 (0.82–2.64)
Neuropathy†	1.04 (0.76–1.44)	—	—
Peripheral artery disease			
Mild to moderate	1.05 (0.84–1.32)	1.48 (1.05–2.09)	3.18 (1.38–7.32)
Severe	1.77 (1.19–2.64)	2.30 (1.35–3.94)	6.72 (2.37–19.0)
Missing data†	1.74 (1.05–2.90)	1.37 (0.88–2.14)	3.96 (1.56–10.0)
Ulcer size (*v* small, < 1 cm^2)			
Medium (1–3 cm^2)	1.10 (0.84–1.45)	1.40 (0.94–2.09)	3.73 (1.37–10.2)
Large (> 3 cm^2)	1.24 (0.95–1.63)	1.21 (0.81–1.79)	3.65 (1.39–9.58)
Missing data†	1.34 (1.02–1.76)	1.46 (0.99–2.15)	4.20 (1.66–10.7)
Infection			
Mild	1.29 (1.02–1.63)	1.36 (0.97–1.90)	—
Moderate to severe	1.47 (1.10–1.96)	1.70 (1.15–2.49)	—
Deep ulcer	1.28 (1.02–1.61)	2.00 (1.45–2.76)	2.08 (1.14–3.80)
Recent DFU treatment provider			
Podiatrist	0.83 (0.55–1.26)	—	—
Surgical specialist	0.99 (0.67–1.46)	0.85 (0.51–1.42)	—
Nurse	0.79 (0.61–1.01)	1.55 (1.14–2.09)	—
Current DFU treatment type			
Debrided ulcer†	0.88 (0.63–1.22)	0.64 (0.43–0.95)	—
Knee-high offloading	0.85 (0.69–1.05)	0.72 (0.55–0.95)	—
Footwear appropriate	0.92 (0.74–1.14)	—	—

CI = confidence interval; DFU: diabetes-related foot ulcer.

* The analyses by outcome category (no amputation, minor amputation, major amputation) include variables for which $P < 0.1$ in univariable analyses (table 4).

† . The variables neuropathy, peripheral artery disease, ulcer size, and debrided ulcer included a missing data category (19–25% missing values).

Table 4. Unadjusted hazard ratios in people with diabetes-related foot ulcers for first diabetes-related foot ulcer-caused hospitalisation with no amputation, minor amputation, and major amputation: univariable Cox hazard regression analyses (censored at 24 months)

	Hazard ratio (95% confidence interval)		
**Characteristic**	**No amputation**	**Minor amputation**	**Major amputation**
**Demographic characteristics**			
Age, per year	0.99 (0.99–1.001)	1.00 (0.99–1.01)	1.01 (0.99–1.04)
Sex (women *v* men)	0.97 (0.83–1.15)	0.88 (0.67–1.16)	1.29 (0.72–2.30)
Indigenous people	1.51 (1.23–1.86)	1.07 (0.73–1.56)	1.92 (0.96–3.85)
Geographic remoteness (*v* major city)			
Regional area	1.15 (0.98–1.35)	1.06 (0.82–1.37)	0.79 (0.44–1.41)
Remote/very remote area	1.47 (1.09–1.98)	1.31 (0.80–2.15)	0.00 (0.00–infinite)
Diabetes type: type 2 (*v* type 1)	0.81 (0.63–1.03)	0.91 (0.60–1.38)	2.34 (0.57–9.64)
Smokes	1.06 (0.84–1.33)	1.40 (1.003–1.95)	0.64 (0.23–1.78)
**Other medical conditions**			
Hypertension	1.11 (0.96–1.30)	1.23 (0.96–1.57)	0.94 (0.54–1.64)
Dyslipidaemia	1.10 (0.95–1.29)	1.21 (0.95–1.55)	1.15 (0.65–2.03)
Cardiovascular disease	1.26 (1.06–1.50)	0.99 (0.73–1.34)	2.12 (1.19–3.77)
Chronic kidney disease	1.21 (0.98–1.49)	1.15 (0.81–1.63)	2.20 (1.15–4.21)
End-stage renal disease	1.29 (0.90–1.84)	2.59 (1.70–3.94)	4.95 (2.32–10.6)
**Limb and ulcer**			
Previous foot ulcer	1.10 (0.92–1.33)	0.94 (0.70–1.25)	1.79 (0.81–3.98)
Previous amputation	1.41 (1.21–1.65)	1.82 (1.42–2.33)	2.30 (1.32–4.00)
Neuropathy	1.09 (0.84–1.41)	1.46 (0.92–2.32)	1.06 (0.42–2.69)
Peripheral artery disease			
Mild to moderate	1.17 (0.97–1.40)	1.71 (1.29–2.27)	4.20 (1.88–9.40)
Severe	1.94 (1.44–2.62)	3.38 (2.23–5.11)	10.25 (3.84–27.4)
Missing data†	1.37 (1.12–1.69)	1.07 (0.72–1.58)	3.80 (1.53–9.45)
Foot deformity	1.11 (0.92–1.34)	1.33 (0.96–1.83)	2.26 (1.04–4.91)
Acute Charcot foot	0.87 (0.47–1.63)	0.68 (0.22–2.13)	0.00 (0.00–infinite)
Ulcer size (*v* small, < 1 cm^2)			
Medium (1–3 cm^2)	1.23 (0.99–1.53)	2.01 (1.42–2.85)	3.77 (1.39–10.2)
Large (> 3 cm^2)	1.61 (1.31–1.97)	2.06 (1.45–2.92)	5.27 (2.04–13.6)
Missing data†	1.28 (1.05–1.57)	1.76 (1.25–2.47)	4.79 (1.90–12.1)
Infection			
Mild	1.36 (1.13–1.63)	1.73 (1.30–2.31)	1.15 (0.59–2.24)
Moderate to severe	2.03 (1.67–2.47)	2.68 (1.97–3.64)	1.41 (0.65–3.08)
Deep ulcer	1.59 (1.33–1.89)	2.85 (2.21–3.68)	2.55 (1.42–4.58)
**Recent DFU treatment provider**			
Podiatrist	0.71 (0.53–0.96)	0.80 (0.48–1.32)	1.25 (0.30–5.16)
General practitioner	0.92 (0.69–1.22)	1.32 (0.88–1.96)	0.73 (0.23–2.35)
Surgical specialist	1.47 (1.13–1.92)	1.86 (1.26–2.76)	0.63 (0.15–2.59)
Medical specialist	1.19 (0.96–1.48)	1.47 (1.07–2.03)	1.10 (0.50–2.46)
Nurse	1.05 (0.88–1.25)	1.55 (1.20–2.01)	1.15 (0.62–2.13)
Other	1.31 (1.05–1.63)	1.12 (0.77–1.63)	1.12 (0.48–2.63)
**Current DFU treatment type**			
Debrided ulcer	0.67 (0.52–0.86)	0.46 (0.32–0.66)	1.23 (0.38–3.98)

Dressing appropriate	0.80 (0.49–1.32)	0.57 (0.28–1.16)	-
Antibiotics prescribed	1.66 (1.42–1.94)	2.32 (1.81–2.98)	0.98 (0.54–1.79)
Knee-high offloading	0.88 (0.76–1.03)	0.78 (0.61–0.998)	1.48 (0.83–2.66)
Footwear appropriate	0.85 (0.72–0.99)	1.00 (0.78–1.29)	1.24 (0.68–2.27)
Patient educated	0.73 (0.32–1.62)	1.55 (0.22–11.1)	-

DFU: diabetes-related foot ulcer.
† The variables neuropathy, peripheral artery disease, ulcer size, and debrided ulcer included a missing data category (19–25% missing values).

Table 5. Adjusted hazard ratios by age in people with diabetes-related foot ulcers, with a reference of 60 years old, for first diabetes-related foot ulcer-caused hospitalisation with no amputation, minor amputation, and major amputation: multivariable flexible parametric survival analyses (censored at 24 months)

Age (years)	No amputation	Minor amputation	Major amputation
25	1.388 (0.772–2.495)	1.144 (0.488–2.679)	0.005 (0.000–0.230)
26	1.384 (0.794–2.412)	1.139 (0.504–2.572)	0.006 (0.000–0.247)
27	1.380 (0.817–2.332)	1.134 (0.520–2.470)	0.008 (0.000–0.265)
28	1.376 (0.839–2.256)	1.129 (0.537–2.374)	0.010 (0.000–0.283)
29	1.371 (0.861–2.184)	1.124 (0.554–2.282)	0.013 (0.001–0.303)
30	1.366 (0.882–2.116)	1.119 (0.571–2.196)	0.016 (0.001–0.324)
31	1.361 (0.903–2.051)	1.115 (0.588–2.113)	0.019 (0.001–0.345)
32	1.355 (0.923–1.990)	1.110 (0.605–2.036)	0.024 (0.002–0.368)
33	1.349 (0.942–1.931)	1.105 (0.623–1.962)	0.029 (0.002–0.392)
34	1.342 (0.960–1.876)	1.101 (0.640–1.892)	0.036 (0.003–0.417)
35	1.335 (0.977–1.824)	1.096 (0.658–1.826)	0.044 (0.004–0.442)
36	1.328 (0.993–1.775)	1.092 (0.676–1.764)	0.054 (0.006–0.469)
37	1.320 (1.007–1.729)	1.087 (0.693–1.705)	0.065 (0.008–0.497)
38	1.311 (1.020–1.686)	1.083 (0.711–1.650)	0.078 (0.012–0.525)
39	1.302 (1.031–1.645)	1.078 (0.728–1.597)	0.094 (0.016–0.554)
40	1.293 (1.041–1.607)	1.074 (0.745–1.548)	0.112 (0.021–0.584)
41	1.283 (1.049–1.570)	1.070 (0.763–1.501)	0.132 (0.029–0.614)
42	1.273 (1.054–1.536)	1.066 (0.779–1.457)	0.156 (0.038–0.644)
43	1.262 (1.059–1.504)	1.062 (0.796–1.416)	0.183 (0.050–0.675)
44	1.251 (1.061–1.474)	1.057 (0.812–1.377)	0.214 (0.065–0.706)
45	1.239 (1.062–1.445)	1.053 (0.828–1.341)	0.248 (0.084–0.737)
46	1.226 (1.061–1.417)	1.049 (0.843–1.306)	0.286 (0.107–0.767)
47	1.213 (1.058–1.391)	1.046 (0.858–1.274)	0.328 (0.135–0.797)
48	1.200 (1.055–1.364)	1.042 (0.872–1.244)	0.373 (0.169–0.826)
49	1.186 (1.051–1.338)	1.038 (0.886–1.216)	0.422 (0.209–0.854)
50	1.171 (1.046–1.311)	1.034 (0.899–1.189)	0.474 (0.255–0.880)
51	1.156 (1.040–1.285)	1.030 (0.912–1.165)	0.529 (0.309–0.905)
52	1.140 (1.034–1.257)	1.027 (0.924–1.142)	0.585 (0.369–0.928)
53	1.124 (1.029–1.229)	1.023 (0.935–1.120)	0.644 (0.436–0.949)
54	1.108 (1.023–1.199)	1.020 (0.946–1.100)	0.702 (0.510–0.967)
55	1.091 (1.018–1.169)	1.016 (0.956–1.081)	0.760 (0.588–0.982)
56	1.073 (1.013–1.137)	1.013 (0.965–1.063)	0.816 (0.670–0.994)
57	1.055 (1.008–1.104)	1.010 (0.974–1.046)	0.870 (0.755–1.002)
58	1.037 (1.005–1.069)	1.006 (0.983–1.030)	0.919 (0.839–1.006)
59	1.018 (1.002–1.035)	1.003 (0.992–1.015)	0.963 (0.922–1.006)
60	1	1	1
61	0.982 (0.966–0.999)	0.997 (0.986–1.008)	1.030 (0.989–1.072)
62	0.965 (0.934–0.998)	0.994 (0.971–1.017)	1.050 (0.972–1.135)
63	0.950 (0.903–0.998)	0.991 (0.957–1.026)	1.062 (0.950–1.187)
64	0.935 (0.875–0.999)	0.988 (0.943–1.035)	1.063 (0.920–1.229)
65	0.922 (0.850–1.001)	0.985 (0.929–1.045)	1.055 (0.884–1.260)
66	0.911 (0.827–1.003)	0.983 (0.914–1.056)	1.038 (0.843–1.279)

Age (years)	No amputation	Minor amputation	Major amputation
67	0.902 (0.808–1.006)	0.980 (0.899–1.068)	1.013 (0.796–1.289)
68	0.895 (0.793–1.010)	0.977 (0.883–1.082)	0.980 (0.744–1.291)
69	0.890 (0.781–1.015)	0.975 (0.867–1.096)	0.940 (0.688–1.285)
70	0.889 (0.773–1.021)	0.972 (0.850–1.112)	0.895 (0.630–1.272)
71	0.889 (0.768–1.029)	0.970 (0.833–1.128)	0.846 (0.571–1.255)
72	0.892 (0.767–1.039)	0.967 (0.816–1.147)	0.795 (0.512–1.233)
73	0.898 (0.768–1.050)	0.965 (0.798–1.167)	0.741 (0.454–1.207)
75	0.906 (0.770–1.065)	0.963 (0.780–1.188)	0.686 (0.399–1.179)
76	0.928 (0.781–1.104)	0.958 (0.743–1.236)	0.578 (0.299–1.117)
77	0.943 (0.787–1.130)	0.956 (0.724–1.263)	0.525 (0.255–1.084)
78	0.960 (0.793–1.161)	0.954 (0.705–1.291)	0.475 (0.215–1.050)
79	0.979 (0.800–1.199)	0.952 (0.686–1.321)	0.428 (0.180–1.016)
80	1.000 (0.806–1.242)	0.949 (0.667–1.352)	0.383 (0.150–0.981)
81	1.024 (0.811–1.294)	0.947 (0.648–1.386)	0.342 (0.124–0.946)
82	1.050 (0.815–1.352)	0.945 (0.629–1.422)	0.304 (0.101–0.912)
83	1.078 (0.819–1.420)	0.943 (0.610–1.459)	0.268 (0.082–0.877)
84	1.109 (0.823–1.496)	0.941 (0.591–1.498)	0.237 (0.066–0.843)
85	1.142 (0.825–1.581)	0.939 (0.573–1.540)	0.208 (0.053–0.809)
86	1.178 (0.827–1.677)	0.937 (0.555–1.583)	0.182 (0.043–0.777)
87	1.216 (0.829–1.784)	0.936 (0.537–1.629)	0.159 (0.034–0.745)
88	1.256 (0.830–1.902)	0.934 (0.520–1.677)	0.138 (0.027–0.713)
89	1.299 (0.830–2.033)	0.932 (0.503–1.726)	0.120 (0.021–0.683)
90	1.344 (0.830–2.177)	0.930 (0.486–1.778)	0.104 (0.017–0.654)

Figure 2. Independent associations between the age of people with diabetes-related foot ulcers (DFU) who visited Queensland Diabetic Foot Service clinics for the first time during 1 July 2011 – 31 December 2017, and first DFU-related hospitalisation outcomes, with a reference of 60 years old: multivariable flexible parametric survival analyses (censored at 24 months)*

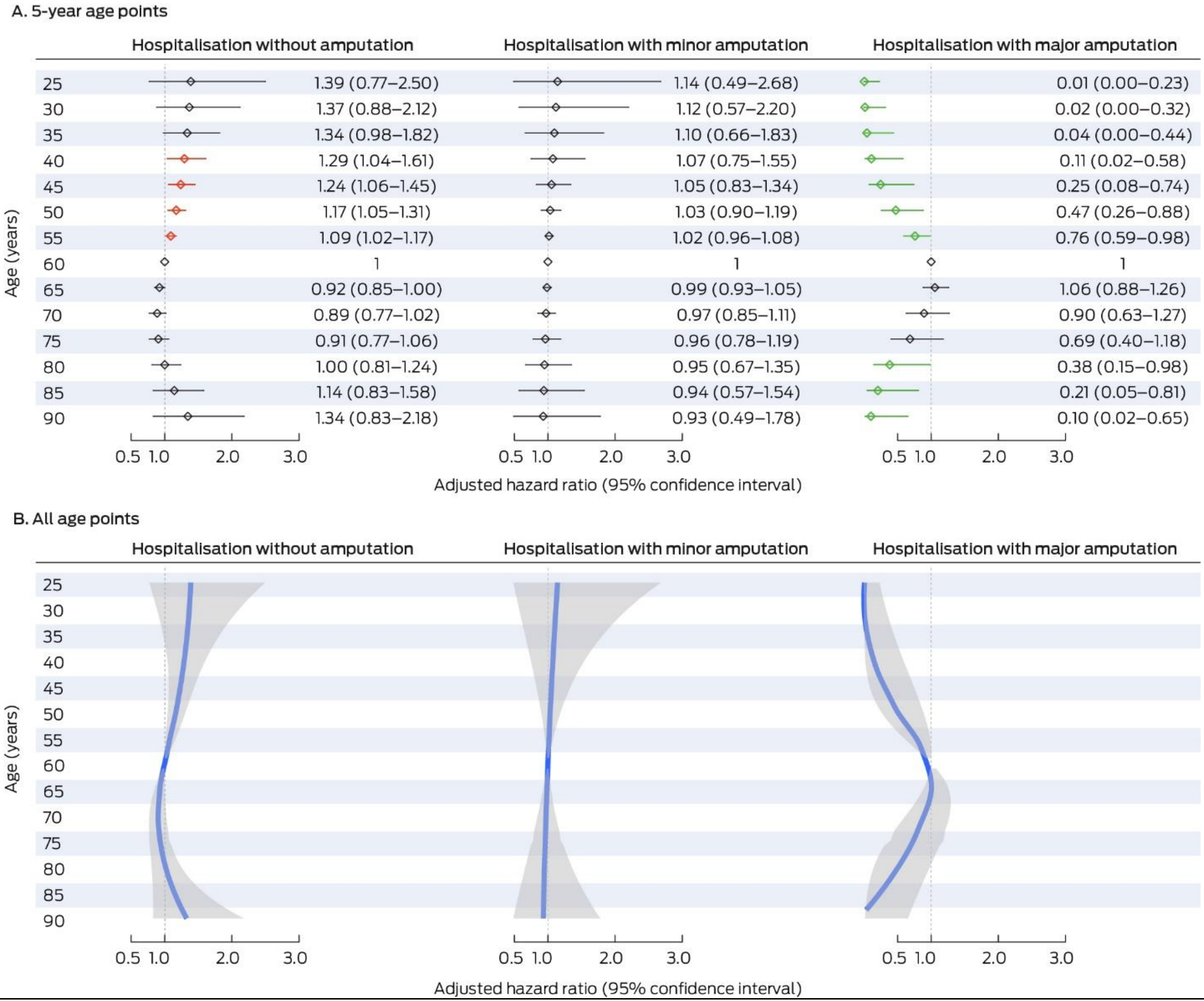

* Each analysis is adjusted for the corresponding variables in Box 3 of the main article. Panel A: Red: increased risk; green: reduced risk; black: no statistically significant difference in risk. Panel B: The adjusted hazard ratios for all ages are depicted as a blue line, the 95% confidence interval as a grey band.

Strengthening the Reporting of Observational studies in Epidemiology checklist

Note: The page numbers in this checklist refer to the submitted manuscript, not to the published article or its Supporting Information file

	Item No	Recommendation	Page location in our study
**Title and abstract**	1	(*a*) Indicate the study's design with a commonly used term in the title or the abstract	Page 1 Title & Page 2: Abstract
		(*b*) Provide in the abstract an informative and balanced summary of what was done and what was found	Page 2: Abstract
**Introduction**			
Background/rationale	2	Explain the scientific background and rationale for the investigation being reported	Page 4: Introduction
Objectives	3	State specific objectives, including any prespecified hypotheses	Page 4: Introduction
**Methods**			
Study design	4	Present key elements of study design early in the paper	Page 4: Study design
Setting	5	Describe the setting, locations, and relevant dates, including periods of recruitment, exposure, follow-up, and data collection	Page 4-5: Participants and settings
Participants	6	(*a*) Give the eligibility criteria, and the sources and methods of selection of participants. Describe methods of follow-up	Page 4-5: Participants and settings; Page 5-6, Outcomes
		(*b*) For matched studies, give matching criteria and number of exposed and unexposed	Not applicable
Variables	7	Clearly define all outcomes, exposures, predictors, potential confounders, and effect modifiers. Give diagnostic criteria, if applicable	Page 5-6: Variables, Outcomes; eTable 2 (Page 2-3 of Supplementary Material)
Data sources/ measurement	8*	For each variable of interest, give sources of data and details of methods of assessment (measurement). Describe comparability of assessment methods if there is more than one group	Page 5-6: Variables, Outcomes; eTable 2 (Page 2-3 of Supplementary Material)
Bias	9	Describe any efforts to address potential sources of bias	Page 6: Statistical analyses
Study size	10	Explain how the study size was arrived at	Page 4-5: Participants and settings; Page 16: Figure 1
Quantitative variables	11	Explain how quantitative variables were handled in the analyses. If applicable, describe which groupings were chosen and why	Page 6: Statistical analyses
Statistical methods	12	(*a*) Describe all statistical methods, including those used to control for confounding	Page 6: Statistical analyses
		(*b*) Describe any methods used to examine subgroups and interactions	Not applicable
		(*c*) Explain how missing data were addressed	Page 6: Statistical analyses
		(*d*) If applicable, explain how loss to follow-up was addressed	Page 5-6, Outcomes
		(*e*) Describe any sensitivity analyses	Not applicable
**Results**			
Participants	13*	(a) Report numbers of individuals at each stage of study—eg numbers potentially eligible, examined for eligibility, confirmed eligible, included in the study, completing follow-up, and analysed	Page 7: Participants; Page 16: Figure 1
		(b) Give reasons for non-participation at each stage	Page 7: Participants; Page 16: Figure 1
		(c) Consider use of a flow diagram	Page 16: Figure 1
Descriptive data	14*	(a) Give characteristics of study participants (eg demographic, clinical, social) and information on exposures and potential confounders	Page 7: Participants; Page 19: Table 1; eTable 3 (Page 6 of Supplementary Material)
		(b) Indicate number of participants with missing data for each variable of interest	Page 19: Table 1
		(c) Summarise follow-up time (eg, average and total amount)	Page 7: Participants
Outcome data	15*	Report numbers of outcome events or summary measures over time	Page 7: Incidence; Page 16: Figure 1; eFigure 1 (Page 7 of Supplementary Material)

Main results	16	(*a*) Give unadjusted estimates and, if applicable, confounder-adjusted estimates and their precision (eg, 95% confidence interval). Make clear which confounders were adjusted for and why they were included	Page 7-8: Risk factors; Page 17- 18: Figures 2-3; Page 19-22: Tables 1-2
		(*b*) Report category boundaries when continuous variables were categorized	Page 17- 18: Figures 2-3
		(*c*) If relevant, consider translating estimates of relative risk into absolute risk for a meaningful time period	Not applicable
Other analyses	17	Report other analyses done—eg analyses of subgroups and interactions, and sensitivity analyses	Not applicable
**Discussion**			
Key results	18	Summarise key results with reference to study objectives	Page 8: Discussion
Limitations	19	Discuss limitations of the study, taking into account sources of potential bias or imprecision. Discuss both direction and magnitude of any potential bias	Page 11: Limitations
Interpretation	20	Give a cautious overall interpretation of results considering objectives, limitations, multiplicity of analyses, results from similar studies, and other relevant evidence	Page 8-10: Discussion
Generalisability	21	Discuss the generalisability (external validity) of the study results	Page 8-9: Discussion
**Other information**			
Funding	22	Give the source of funding and the role of the funders for the present study and, if applicable, for the original study on which the present article is based	Stated in Scholar One submission system

*Give information separately for exposed and unexposed groups.

Note: An Explanation and Elaboration article discusses each checklist item and gives methodological background and published examples of transparent reporting. The STROBE checklist is best used in conjunction with this article (freely available on the Web sites of PLoS Medicine at http://www.plosmedicine.org/, Annals of Internal Medicine at http://www.annals.org/, and Epidemiology at http://www.epidem.com/). Information on the STROBE Initiative is available at http://www.strobe-statement.org.
